# Radiomics nomogram based on MRI water imaging identifying symptomatic nerves of patients with primary trigeminal neuralgia: A preliminary study

**DOI:** 10.1097/MD.0000000000037379

**Published:** 2024-03-01

**Authors:** Hongjian Li, Chuan Zhang, Wei Yan, Zeyong Li, Ying Liu, Baijintao Sun, Libing He, Qimin Yang, Xu Lang, Xiran Shi, Ting Lei, Anup Bhetuwal, Hanfeng Yang

**Affiliations:** a Department of Radiology, Affiliated Hospital of North Sichuan Medical College, Nanchong, People’s Republic of China; b Department of Radiology, Bishan Hospital of Chongqing Medical University, Chongqing, People’s Republic of China; c The First Affiliated Hospital of Chengdu Medical College, Chengdu, People’s Republic of China.

**Keywords:** logistic regression, MRI, nomogram, radiomics, ROC curve, trigeminal neuralgia

## Abstract

The study proposes a combined nomogram based on radiomics features from magnetic resonance neurohydrography and clinical features to identify symptomatic nerves in patients with primary trigeminal neuralgia. We retrospectively analyzed 140 patients with clinically confirmed trigeminal neuralgia. Out of these, 24 patients constituted the external validation set, while the remaining 116 patients contributed a total of 231 nerves, comprising 118 symptomatic nerves, and 113 normal nerves. Radiomics features were extracted from the MRI water imaging (t2-mix3d-tra-spair). Radiomics feature selection was performed using L1 regularization-based regression, while clinical feature selection utilized univariate analysis and multivariate logistic regression. Subsequently, radiomics, clinical, and combined models were developed by using multivariate logistic regression, and a nomogram of the combined model was drawn. The performance of nomogram in discriminating symptomatic nerves was assessed through the area under the curve (AUC) of receiver operating characteristics, accuracy, and calibration curves. Clinical applications of the nomogram were further evaluated using decision curve analysis. Five clinical factors and 13 radiomics signatures were ultimately selected to establish predictive models. The AUCs in the training and validation cohorts were 0.77 (0.70–0.84) and 0.82 (0.72–0.92) with the radiomics model, 0.69 (0.61–0.77) and 0.66 (0.53–0.79) with the clinical model, 0.80 (0.74–0.87), and 0.85 (0.76–0.94) with the combined model, respectively. In the external validation set, the AUCs for the clinical, radiomics, and combined models were 0.70 (0.60–0.79), 0.78 (0.65–0.91), and 0.81 (0.70–0.93), respectively. The calibration curve demonstrated that the nomogram exhibited good predictive ability. Moreover, The decision curve analysis curve indicated shows that the combined model holds high clinical application value. The integrated model, combines radiomics features from magnetic resonance neurohydrography with clinical factors, proves to be effective in identify symptomatic nerves in trigeminal neuralgia. The diagnostic efficacy of the combined model was notably superior to that of the model constructed solely from conventional clinical features.

## 1. Introduction

Primary trigeminal neuralgia (PTN) is a prevalent form of neuropathic pain in the brain, characterized by recurrent episodes of severe pain in the unilateral facial trigeminal nerve distribution. The lifetime prevalence of PTN is approximately 0.16% to 0.3%, with an annual incidence ranging from 12.6 to 27.0 per 100,000 people-years. The incidence is 3 times higher in female than male,^[1–3]^ and the average age of onset is typically between 53 and 57 years.^[4,5]^ PTN, constituting 75% of trigeminal neuralgia (TN), poses challenges in diagnosis due to its unclear etiology and the absence of diagnostic methods compared to secondary TN, which is typically associated with tumors, inflammation, and other identifiable factors, making it relatively easier to diagnose. The prevailing neurovascular compression (NVC) theory, explaining PTN, is controversial, as not all patients exhibit signs of vascular compression on imaging. This controversy underscores the inadequacy of relying solely on NVC for a comprehensive diagnosis of TN.^[4,6–8]^

The demyelination of trigeminal nerve roots, secondary to pulsatile compression from surrounding microvasculature, is currently considered the primary cause of PTN.^[9]^ Demyelination can lead to dysregulation of voltage-gated sodium channels (Nav), contributing to pain.^[9,10]^ Additionally, clinical features such as morphological changes in the Merkel cavity (MC), reduced trigeminal pons angle (TPA) and atrophic thinning of affected nerves may play roles in PTN etiology.^[11–13]^ Microscopic factors, such as dysregulated expression of long-stranded noncoding RNA, micro-demyelination due to arachnoid adhesions, micro-cholesteatoma, and narrowing of the foramen ovale are also implicated.^[14–16]^ Current diagnostic methods primarily rely on assessing NVC, but this approach is subjective and influenced by physician interpretation, making an objective and accurate diagnostic method crucial.

The field of radiomics, involves the extracting high-throughput features from medical imaging data, utilizing the grayscale variation of images and the spatial arrangement of voxel. This process captures and quantifies the heterogeneity within a lesion or region of interest. Radiomics information not only correlates with the biological state of the disease but also shows promise in characterizing various diseases. While currently widely used in oncology research, its application in pain-related diseases and neurological disorders, including PTN, is limited. This study aims to bridge this gap by combining radiomics with clinical features to create an integrated model for PTN diagnosis. Additionally, clinical features such as TPA, although challenging for visual assessment, can be accurately measured and incorporated into clinical models. However, research in this area is relatively scarce which is why a combined model for diagnosing PTN represents a meaningful and innovative attempt.

Radiomics studies necessitate a substantial sample size of patients and high-resolution images. However, the incidence of TN is low, resulting in a limited number of radiomics studies on PTN. Some existing studies indicate variations in radiomic characteristics of PTN. For instance, patients exhibited lower-than-normal flatness in Meckel cavity, with generally reduced flatness on the right compared to the left.^[17]^ Additionally, certain researchers applied 3D U-Net for the automated segmentation of the intracranial segment of the trigeminal nerve. They affirmed radiomic differences in first-order features and textural features of symptomatic trigeminal nerve.^[18]^ However, these studies faced limitations, such as the exclusion of clinical features, insufficient sample sizes, or the absence of model construction. Hence, our study aimed to address these gaps by incorporating multiple clinical factors associated with TN; utilizing high-resolution magnetic resonance imaging (MRI) images to enhance the precision of radiomic features; ensuring an adequate sample size.

## 2. Materials and methods

### 2.1. Patients

The Institutional Review Board of the Hospital of North Sichuan Medical College approved this retrospective study and waived the requirement for informed consent. Our study adhered to the Declaration of Helsinki. A total of 116 patients with TN diagnosed from January 2020 to June 2023 were retrospectively collected. Since most patients had 1 symptomatic and the other asymptomatic of the 2 trigeminal nerves, they were included in the positive and negative groups, respectively. Therefore, certain clinical characteristics of the bilateral nerves such as gender and age, were excluded due to originating from the same patient. One nerve was excluded due to poor image quality, resulting in a total of 231 nerves included in 116 patients, comprising 117 symptomatic nerves and 114 normal nerves. An additional 24 patients from August to October 2023 were collected as an external validation set, with 24 symptomatic nerves and 24 normal nerves.

Inclusion criteria were MRI completed before surgery; the MRI sequence was t2-mix3d-tra-spair heavy t2 water imaging; patients clinically diagnosed with TN, with PTN diagnosis based on the Third edition of the International Classification of Headache Disorders.^[19]^ Exclusion criteria were the patient’s lesioned nerve is a peripheral branch of the trigeminal nerve, such as pterygopalatine neuralgia and supraorbital neuralgia; the left and right sides affected by TN cannot be determined; patients have undergone surgical treatment such as microvascular compression surgery, percutaneous balloon compression, percutaneous balloon compression (PBC), etc; the images do not meet the analysis requirements: unclear display and artifacts; other water imaging sequences utilized, such as Siemens CISS sequence, GE fiesta-c sequence. Patients with other diseases that may trigger TN, such as tumors in the pontocerebellar angle (meningioma, trigeminal nerve sheath tumor, etc), tumors in the Meckel cavity, multiple sclerosis, etc.

**Figure 1.**
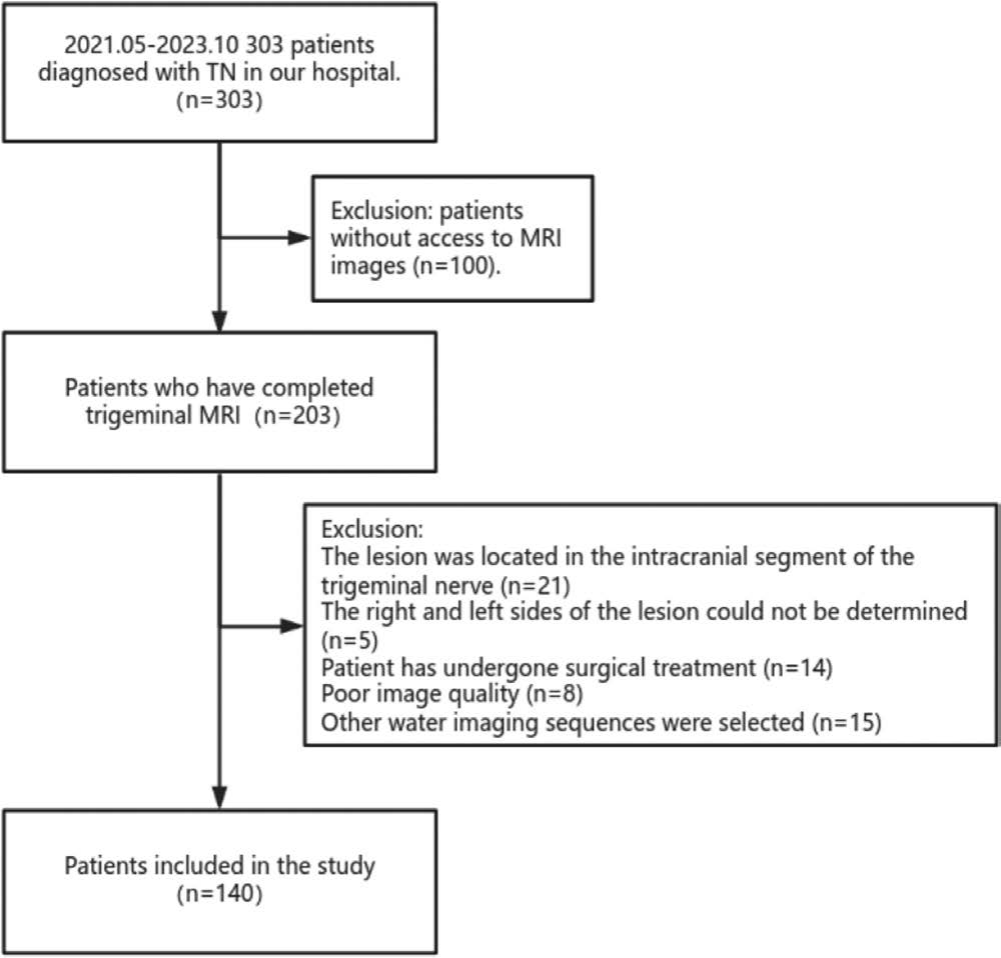
Flowchart of patient recruitment for this study. TN = trigeminal neuralgia.

**Figure 2.**
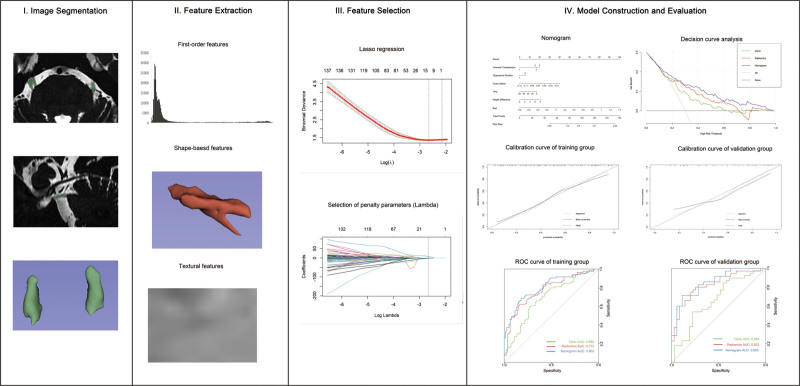
Workflow chart of necessary steps in this study. AUC = area under the curve, ROC = receiver operating characteristic.

The flowchart of this study is presented in Figure 1. The internal validation set was randomly divided in a 7:3 ratio, with 231 nerves categorized into 2 cohorts: a training cohort (n = 162) and a validation cohort (n = 69). All cases in the training cohort were used to construct the prediction models, while the cases in the validation cohort were used to independently evaluate the models’ performance. Groups were divided on a nerve-by-nerve basis rather than a patient-by-patient basis. If a patient exhibited symptoms on only 1 side of the trigeminal nerve, the affected nerve of that patient was included in the symptomatic nerve group, and the nerve on the other side was included in the normal nerve group. The flowchart of this study is illustrated in Figure 2.

### 2.2. MRI scanning protocol

All patients underwent examination with a 3.0T MRI (United Imaging, Discover uMR 790). Trigeminal (t2-mix3d-tra-spair) data were acquired using the following parameters: TR = 1300 millisecond; TE = 258 millisecond; field of view = 180 × 200 mm; slice thickness = 0.5 cm; 74 slices; voxel size = 0.5 mm × 0.5 mm × 0.5mm.

### 2.3. Imaging segmentation and radiomics feature extraction

Two radiologists, each with over 10 years of experience in neuroimaging, independently analyzed the images without knowledge of the patients’ clinical results and reached a consensus. Manual segmentation of axial images was performed using 3D slicer software (version 5.2.1, http://www.slicer.org). The region of interest encompassed the trigeminal nerve from the beginning of the brainstem to the entrance of Merkel cave, carefully avoiding surrounding vessels and forming a volume of interest. Interobserver reproducibility was assessed by the 2 radiologists, who analyzed 20 randomly selected volume of interest images and the intra-group correlation coefficients (ICC) were used to evaluate interobserver agreement of radiomic feature measures.

To mitigate the impact of image factors on radiomic features, a preprocessing step was conducted on the MR images before the radiomic feature extraction process. All images were resampled to a voxel size of 1 × 1 × 1 mm^3^ using B-spline interpolation. Each MRI scan was normalized to achieve a standard normal distribution of image intensities. A total of 1316 radiomics features in 8 categories were extracted using 3D slicer’s radiomics plugin: first-order features, gray-level cooccurrence matrix, gray-level dependence matrix features, gray-level run-length matrix features, gray-level size zone matrix (GLSZM) features, neighborhood gray-tone difference matrix features, 3D shape features, and 2D shape features.

### 2.4. Selection of radiomics features

The extracted features undergo z-score normalization, and features with ICCs >0.75 are retained. Feature selection is performed using least absolute shrinkage and selection operator (LASSO) regression, with the optimal *λ* selected through 10-fold cross-validation, and the corresponding coefficients of the features are calculated. Following the screening of radiomic features, the Radscore score is calculated using the formula: radiomic features × coefficient + intercept.

### 2.5. Conventional MRI evaluation

There are 4 grades of NVC: grade I: cerebrospinal fluid is present between the trigeminal nerve and the blood vessel. Grade II: contact between the trigeminal nerve and the responsible vessels, with no signal shadow of cerebrospinal fluid between them. Grade III: the responsible vessel is compressing the trigeminal nerve, and there is a significant indentation on the nerve root. Grade IV: heavy compression of the nerve root by the responsible vessel, causing displacement and significant atrophy of the nerve.^[20]^ For the analysis, grade I is treated as NVC negative, while grades II, III, and IV are considered NVC positive.^[21]^ Measurements were taken for the bilateral angle of the petrous ridge (APR) and trigeminal nerve angles (ATN) in the sagittal position, APR was measured along the superior edge of the petrous bone to determine the sharpness of its bony ridge. In the sagittal position, ATN was measured along the path from the Meckel cavity to the superior edge of the petrous bone and from the superior edge of the petrous bone to the brain bridge to determine the angle of the trigeminal nerve over the petrous bone ridge.^[20]^ The TPA is measured as follows: in cross-section, TPA consists of 2 lines—the trunk of the trigeminal nerve and the point from which the trigeminal nerve emanates—and the tangent line produced by the cerebral bridge.^[22]^ The location of neurovascular compression (proximity to) was divided into proximal and distal segments. The proximal segment represents half of the trigeminal cerebral pool segment near the cerebral bridge, and the distal segment represents the other half near Meckel cavity.

### 2.6. Clinical feature collection and selection

**Figure 3.**
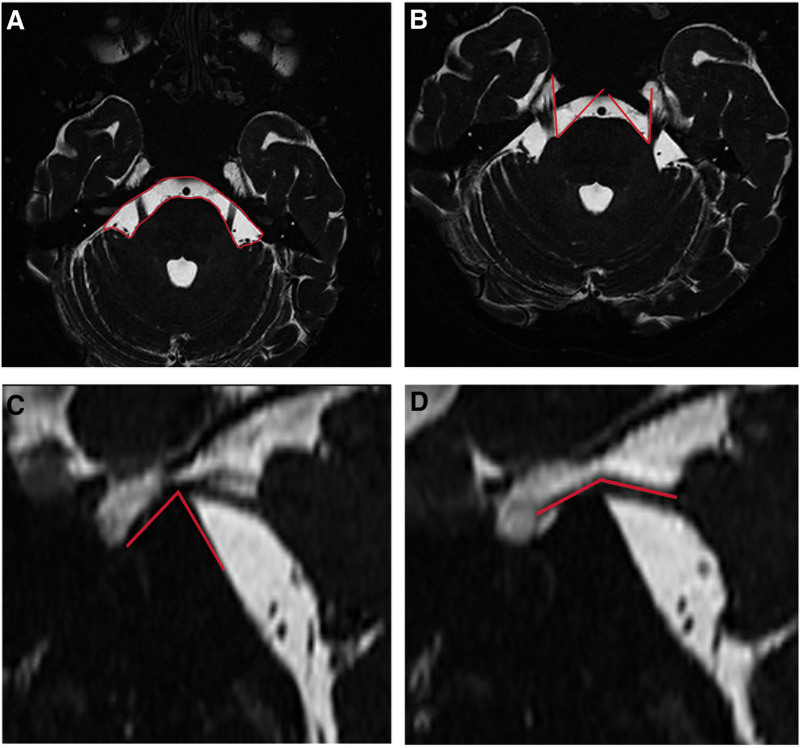
Measurement of some clinical features. (A) The scope and area of the pool in front of the bridge. (B–D) Measurement of TPA, APR, ATN. APR = angle of the petrous ridge, ATN = trigeminal nerve angles, TPA = trigeminal pons angle.

The study encompassed general clinical data, including the degree of vascular compression (divided into 4 levels: away from, in contact with, squeezing, nerve displacement), site of vascular compression (proximity to) of nerves (proximal and distal segments), thickness of adjacent blood vessels, maximum cross-diameter, diameter, cross-sectional area of MC, maximum cross-sectional area of the trigeminal nerve brain pool segment, height difference between the highest and lowest point of the trigeminal nerve brain pool segment, area of the pool in front of the bridge, APR, TPA, and ATN—totaling 13 clinical features. Essential clinical features were measured, as shown in Figure 3.

### 2.7. Model construction and assessment

R language software was employed for model building. Two multifactorial logistic regression models were developed using the described radiomic features and clinical features. Receiver operating characteristic (ROC) curves were plotted to evaluate model performance. Various metrics, including accuracy, specificity, sensitivity, positive predictive value, negative predictive value, Youden index, and area under the curve (AUC) were calculated to identify nerves with TN symptoms. After determining Radscore values for each case, a logistics-based Radscore model was built using the optimal subset of features from the training cohort. Radscore values were integrated with clinical characteristics to establish a multifactorial logistic regression model (combined model) and draw a nomogram for identifying nerves with TN symptoms. Decision curve analysis (DCA) curves were employed to assess model’s clinical application value. Calibration curves were used to evaluate the model’s performance in identifying symptomatic nerves. Corrected AUCs were calculated to address overfitting bias using bootstrapping validation (1000 bootstrap resamples) in the training cohort. The features were then utilized to model the external validation set, and the corresponding ROC curves, calibration curves, and DCA curves were plotted.

### 2.8. Statistical analysis

Statistical analysis was conducted using R language software and SPSS software (version 26.0, IBM Corp., Armonk, NY). Nominal variables were analyzed using the Chi-square test or Fisher exact test while continuous variables with abnormal distributions were analyzed using Mann–Whitney test, and those with normal distributions were analyzed using the *t* test. Univariate and multivariate logistic regression analyses were performed with SPSS software. Clinical factors associated with symptomatic trigeminal nerve were screened with univariate logistic regression analysis at *P *< .05. Independent risk factors were screened with multivariate logistic regression analysis at *P *< .05. The “glmnet” package was utilized for LASSO regression analysis. The“rms” package was used for nomogram construction and calibration plot, the “dcurves” for DCA, and the “pROC” package for plotting ROC curves. DeLong test was used to compare the differences of each model and between the training and validation groups. *P* < 0.05 was considered statistically significant.

## 3. Results

### 3.1. General clinical data

Table 1 displays the baseline clinical characteristics of the normal and symptomatic nerve groups. The normal nerve group exhibited larger maximum cross-sectional area of the trigeminal nerve cistern segment, greater values for TPA, APR, ATN, and the thickness of the responsible vessels compared to the symptomatic nerve group. Conversely, the vertical height of trigeminal nerve cistern segment and the degree of vascular compression were smaller in the normal nerve group. The site of NVC (proximity) in the normal group was predominantly distal to the trigeminal brain pool segment. Other clinical characteristics did not significantly different between the 2 groups.

**Table 1:** Clinical characteristics of patients.

	Overall	Normal nerves	Symptomatic nerves	*P*
N	231	113	118	
Age	62.68 (12.68)	—	—	—
Sex (F/M)	137/94 (59.3/40.7)	—	—	—
Maximum diameter of Mel chamber (mean [SD])	13.24 (2.22)	13.43 (2.21)	13.06 (2.23)	.2
Maximum transverse diameter of Mel chamber (mean [SD])	5.17 (1.05)	5.29 (1.11)	5.06 (0.98)	.097
Maximum cross-sectional area of the Mel cavity (mean [SD])	0.58 (0.18)	0.59 (0.18)	0.56 (0.18)	.147
The maximum cross-sectional area of the trigeminal nerve cistern segment (mean [SD])	509.72 (282.52)	567.42 (295.88)	454.46 (258.46)	.002
Length of trigeminal nerve cistern segment (mean [SD])	11.22 (2.12)	11.37 (2.04)	11.08 (2.18)	.309
TPA (mean [SD])	48.70 (11.78)	49.80 (11.46)	47.64 (12.04)	.035
APR (mean [SD])	100.43 (14.95)	105.17 (14.49)	95.90 (13.99)	<.001
ATN (mean [SD])	144.66 (14.89)	147.94 (14.19)	141.53 (14.93)	.001
Area of the anterior pool of the cerebral bridge (mean [SD])	2.19 (0.66)	2.18 (0.64)	2.19 (0.68)	.952
Vertical height of trigeminal nerve brain pool segment (mean [SD])	2.45 (1.45)	2.23 (1.38)	2.65 (1.48)	.025
Degree of vascular compression (%)				.013
1	42 (18.2)	29 (25.7)	13 (11.0)	
2	53 (22.9)	27 (23.9)	26 (22.0)	
3	88 (38.1)	40 (35.4)	48 (40.7)	
4	48 (20.8)	17 (15.0)	31 (26.3)	.046
Site of vascular compression (proximity to) of nerves = 0/1 (%) (0 for near segment, 1 for far segment)	114/117 (49.4/50.6)	48/65 (42.5/57.5)	66/52 (55.9/44.1)	
Thickness of responsible blood vessels (%)				<.001
1	44 (19.0)	33 (29.2)	11 (9.3)	
2	137 (59.3)	64 (56.6)	73 (61.9)	
3	50 (21.6)	16 (14.2)	34 (28.8)	

APR = angle of the petrous ridge, ATN = trigeminal nerve angles, SD = standard deviation, TPA = trigeminal pons angle.

### 3.2. Traditional assessment of the symptomatic nerves

Univariate analysis identified several clinical features associated with symptomatic nerves, including the maximum cross-sectional area of the trigeminal nerve cistern segment, vertical height of the trigeminal nerve cistern segment, ATN, APR, TPA, site of vascular compression (proximity to) of nerves, degree of vascular compression (*P *< .05). Multivariate logistic regression analysis identified independent risk factors for symptomatic nerves: the maximum cross-sectional area of the trigeminal nerve cistern segment (OR = 0.18; 95% confidence interval [CI], 0.05–0.642), vertical height of trigeminal nerve cistern segment (OR = 1.267; 95% CI, 1.027–1.562, TPA (OR = 0.982 95% CI, 0.959–1.007), site of vascular compression (proximity to) of nerves (OR = 1.873; 95% CI, 1.069–3.283), degree of vascular compression (take degree 4 as reference, degree 1 OR = 0.257; 95% CI, 0.102–0.651, degree 2 OR = 0.548 95% CI, 0.233–1.288, degree 3 OR = 0.693; 95% CI, 0.318–0.511).

### 3.3. Radiomics analysis of the symptomatic nerves, diagnostic performance of the nomogram

**Figure 4.**
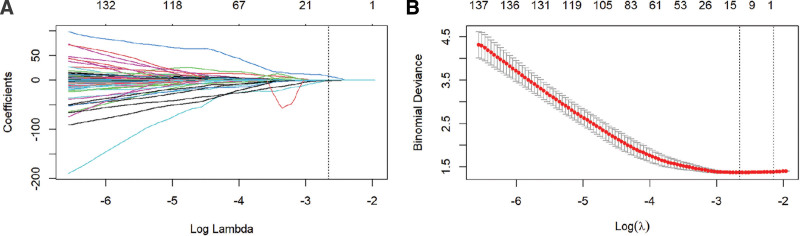
(A) LASSO coefficient convergence of the radiomics features. (B) Penalty parameter (λ) selection using 10-fold cross-validation via criteria of minimum partial deviation variance. The dotted vertical line represents the optimal value of penalty parameter (log (λ) = −2.658) chosen by the minimum deviation variance criteria, resulting in 13 features with nonzero coefficients. LASSO = least absolute shrinkage and selection operator.

A total of 1316 radiomics features were extracted, encompassing 14 shape features, 252 features with first-order statistics, 336 gray scale co-generation matrix features, 196 gray-level dependence matrix features, 224 gray scale tour matrix features, 224 full forms (GLSZM) features, and 70 full forms (NGDTM) features. Out of the 1316 extracted features, 1160 demonstrated high stability. The ICC (intra) and ICC (inter) ranges for all radiomics features were 0.700 to 0.905 and 0.688 to 0.873, respectively, indicating good inter- and intra-reproducibility. Following the application of the LASSO algorithm, 13 features were ultimately retained and utilized for model construction (see Fig. 4 for details), including shape-sphericity, first-order-maximum, GLSZM-large area high gray, level emphasis, and other radiomics features (refer to the Appendix for details, Supplemental Digital Content, http://links.lww.com/MD/L828). The AUC values, specificity, sensitivity, accuracy, and Youden index of the radiomics model in both the training and validation groups surpassed those of the clinical model (Table 2). Differences between each model were assessed using the Delong test, revealing significantly higher AUC values for the combined model compared to the clinical model (*P* = .023 for the training group and *P* = .034 for the validation group). However, no significant improvement in diagnostic efficacy was observed for the combined model compared to the radiomics model.

**Table 2:** Performance of the clinical model, radiomics model, and clinical-radiomics model in the training cohort and test cohort.

Training cohort/validation cohort			
	Clinical model	Radiomics model	Nomogram
AUC	0.69 (0.61–0.77)/0.66 (0.53–0.79)	0.77 (0.70–0.84)/0.82 (0.72–0.92)	0.80 (0.74–0.87)/0.85 (0.76–0.94)
ACC	0.65 (0.57–0.72)/0.60 (0.48–0.72)	0.71 (0.63–0.78)/0.74 (0.62–0.84)	0.72 (0.64–0.89)/0.76 (0.64–0.85)
SEN	0.66 (0.54–0.76)/0.64 (0.47–0.78)	0.67 (0.56–0.77)/0.69 (0.51–0.83)	0.73 (0.61–0.81)/0.83 (0.66–0.93)
SPE	0.64 (0.52–0.74)/0.56 (0.36–0.73)	0.75 (0.64–0.84)/0.80 (0.63–0.92)	0.72 (0.60–0.81)/0.80 (0.63–0.92)
PPV	0.64 (0.53–0.74)/0.64 (0.47–0.79)	0.73 (0.62–0.83)/0.77 (0.59–0.90)	0.73 (0.62–0.82)/0.73 (0.56–0.85)
NPV	0.66 (0.54–0.76)/0.55 (0.36–0.73)	0.69 (0.58–0.78)/0.72 (0.55–0.85)	0.71 (0.60–0.81)/0.80 (0.61–0.92)
YI	0.3/0.2	0.42/0.43	0.45/0.53

ACC = accuracy, AUC = area under the curve, NPV = negative predictive value, PPV = positive predictive value, SEN = sensitivity, SPE = specificity, YI = Youden index.

**Figure 5.**
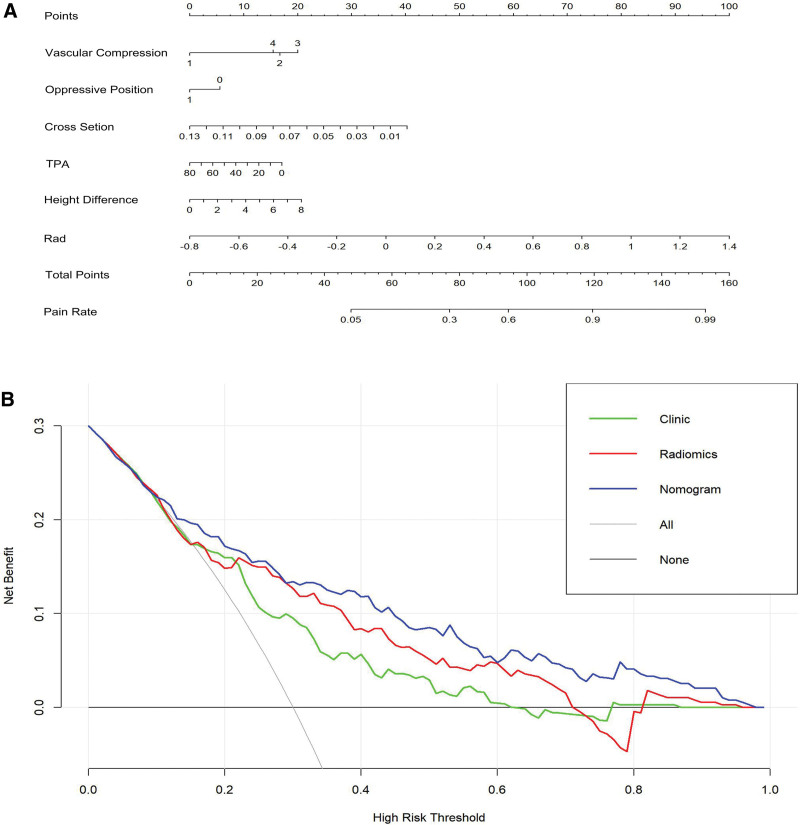
(A) Nomogram for identifying symptomatic trigeminal nerve. (B) Decision curve analysis of radiomics signature, clinical model, and nomogram, respectively. TPA =  trigeminal pons angle.

**Figure 6.**
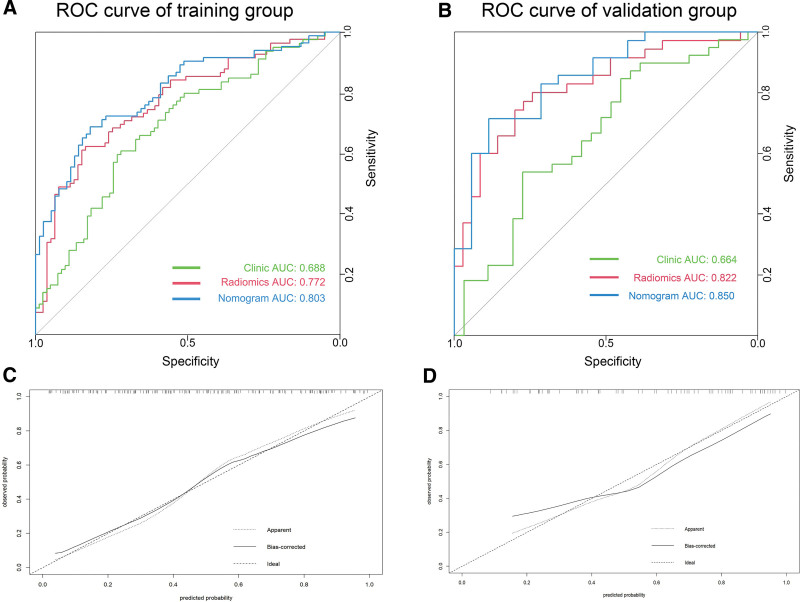
(A, B) Receiver operating characteristic curve analysis in the training group and validation group. (C, D) The nomogram calibration was assessed using calibration curves, confirmed by Hosmer–Lemeshow test in the training and validation sets. AUC = area under the curve, ROC = receiver operating characteristic.

**Figure 7.**
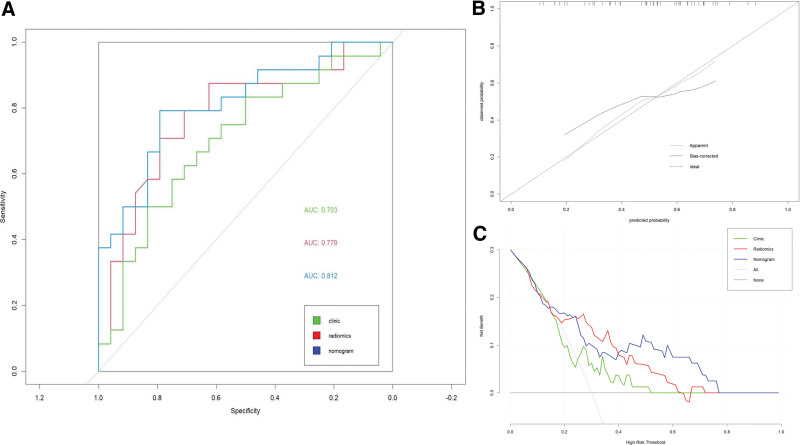
(A) ROC curves for the external validation set with AUC values of 0.70, 0.78, and 0.81 for its clinical model, radiomics model, and combined model, respectively. (B) Calibration curves for external validation sets. (C) DCA curves for external validation sets. AUC = area under the curve.

Table 2 displays the AUC values, accuracy, and corresponding 95% CIs of the combined model based on clinical characteristics and Rad score. By integrating radiomic and clinical characteristics, the AUC values of the combined model improved to 0.80 (training group, 0.74–0.87), and 0.85 (validation group, 0.76–0.94). Figures 5 and 6 illustrate the ROC and calibration curves. The AUC for the external validation set reached 0.81 (0.70–0.93), significantly surpassing the clinical model (*P* = .031) and marginally outperforming the radiomics model, consistent with the internal validation set. The ROC curves, calibration curves, and DCA curves for the external validation set are depicted in Figure 7.

## 4. Discussion

In recent years, with the advancement of modern medical imaging, multimodal MRI examinations have enhanced the accuracy of diagnosing NVC-positive. Approximately 3% of TN cases are classified as secondary TN. These instances are frequently associated with identifiable cause such as tumors, multiple sclerosis, posterior cranial fossa stenosis, Meckel cavity stenosis, and other causative factors, making them relatively straightforward to diagnose.^[23]^ However, some PTN’s without NVC may be linked to microscopic secondary factors. Conditions such as arachnoid adhesions, microscopic arteriovenous compression, nonorganic damage, and functional alterations in the trigeminal nerve within the cerebral pool segment (e.g., a significant increase in the mean creatine concentration of the nerve in the cerebral pool segment) pose challenges in terms of diagnosis.^[16,24]^ Hence, this study utilized radiomics to extract the radiomic features of symptomatic and normal nerves. By integrating these features with clinical characteristics, we established an auxiliary diagnostic model for TN, demonstrating excellent diagnostic efficacy.

Several studies have indicated a potential association between trigeminal root atrophy and pain production, whether or not vascular compression is present. In other words, atrophy of the trigeminal roots can initiate demyelination changes that subsequently result in sodium channel imbalance. This pathophysiological model offers an explanation for PTN caused by various factors, including genetic and idiosyncratic factors, even in the absence of NVC.^[13,25,26]^ Our research aligns with the aforementioned research conclusions. The key variables prominently featured in the clinical model are the maximum cross-sectional area of the trigeminal nerve pool segment and NVC. Across both the training and validation groups, the combined model, integrating radiomics and clinical variables, demonstrated notable enhancements in diagnostic performance and AUC values. Notably, when eliminating the variables associated with the maximum cross-sectional area of the trigeminal nerve and vascular compression, the corresponding combined model exhibited minimal improvement in diagnostic performance. This suggests that atrophy and thinning of the pool segment may represent the most significant morphological variables in TN, with NVC being its primary inducement. This comparison also implies that the radiomic model does not comprehensively encompass its main clinical features. We posit that the thinness of the trigeminal nerve, akin to the minimum diameter thickness for outlining the target area, might be a contributing factor. If the trigeminal nerve thickness falls below the minimum diameter of the outlined target area, its thickness is treated as equal to this minimum diameter, failing to accurately reflect the degree of trigeminal atrophy. Additionally, limiting the target area to the cerebral pool segment of the nerve does not accurately reflect the degree of NVC, as smaller indentation and nerve displacements are challenging to identify within this isolated nerve target area. This situation underscores that, despite the radiomics model having a broad coverage of other clinical indicators, it may not adequately capture the 2 clinical indicators mentioned above. The radiomic model seems to be well-weighted for other reliable predictors, emphasizing its potential of radiomics in identifying the affected nerve, particularly for those affected nerve without NVC.

In this study, 111 (94%) symptomatic nerves and 58 (51%) normal nerves exhibited NVC, with the symptomatic nerves being finer than the normal nerves. This highlights that NVC is not a necessary condition for PTN and suggests that higher levels of NVC may be more likely to lead to demyelination and nerve atrophy. A meta-analysis of high-quality blinded and controlled studies revealed that 471 of 531 symptomatic nerves (89%) and 244 of 681 asymptomatic nerves (36%) had neurovascular contacts.^[27]^ Our study reported a higher rate of NVC positivity compared to this analysis. The rationale for this difference is that our study excluded secondary TN caused by tumors, multiple sclerosis, and so on, and retained more cases of vascular compression type PTN. It is noteworthy that, since our study employed asymptomatic nerves of PTN patients as the normal nerve group, it raises the question of whether normal trigeminal nerves in PTN patients are more prone to NVC than those in normal individuals Bilateral NVC typically manifests symptoms only on the severe side, and the degree of NVC in asymptomatic nerves may not be sufficient to cause demyelination and nerve root atrophy. These subtle distinctions may only be discerned through radiomics methods. Additionally, Holste et al^[28]^ and Amaya et al^[29]^ have indicated that the type of responsible vessel may also be a pathophysiological factor in causing PTN and a predictor of the efficacy of microvascular decompression.^[30]^ In other studies,^[31]^ patients with NVC located close to the root or proximal segment of the trigeminal nerve were more prone to TN, aligning with the results of the present study. This can be explained by the myelin in this segment being part of the central-to-peripheral transition and more susceptible to demyelination. Zhao et al^[32]^ similarly used NVC classification and compression location to establish a diagnostic model for TN, yielding positive outcomes.

Our findings revealed that a decrease in TPA was also identified as a predictor of PTN. Ha et al^[33]^ proposed that a smaller TPA was more likely to result in adhesions between the trigeminal root and the arachnoid around the cerebral bridge, increasing the likelihood of contact with blood vessels. However, contrary to our study previous research^[34,35]^ suggested that APR and ATN could serve as predictors of TN In our study, they did not emerge as effective predictors, possibly due to age-related osteophytic changes causing bone spurs to become sharper, and resulting decreased APR and ATN.^[36]^ Age might play a role in the onset of TN, emphasizing the need for a stratified analysis of patients within the same age group or with similar degree of bone hyperplasia, but the lack of appropriate criteria for patients grouping limits this investigation.^[7]^ The vertical height of the trigeminal brain pool segment was another predictor variable in our study, though it is not currently reported in the literature. This variable was included based on our observation that the vertical height of symptomatic nerves appeared generally higher when outlining the neural target areas. We speculate that a greater vertical height makes it easier for nerves in the cerebral pool segment to come into contact with more levels of vascular compression and for the nerves themselves to bend and even fold. MC is also a crucial pathway for the trigeminal nerve, and previous studies have suggested that morphological atrophy of the MC can lead to narrowing of the cavity space, potentially causing compression of the trigeminal nerve in the MC segment and resulting in TN.^[37]^ However, the parameters related to MC size were not sufficient as predictors in this study. We believe that the probability of MC morphological atrophy in PTN patients is low, rendering it an insufficient predictor. Additionally, in the treatment of PTN through PBC, the shape characteristics of MC are also vital factors affecting the efficacy and recurrence of PBC. Thus, multiple anatomical characteristics of MC may influence PTN, not limited to size alone.^[38]^ Lin et al^[17]^ found histological differences in the MC morphology of PTN patients and healthy individuals through radiomics studies, although these differences have not been utilized in diagnostic modeling.

The study has certain shortcomings and limitations. First, as a radiomics study, the number of patients is not yet large, and efforts are ongoing to collect more patients and strive for potentially improved results. Second, the target area outline of radiomics was restricted to the trigeminal nerve in the cerebral pool segment. In addition to the MC, PTN patients exhibit alterations in other areas, such as changes in the volume, atrophy, and gyrification of the gray matter in the brain, including the precentral gyrus, middle temporal gyrus, postcentral gyrus, midbrain aqueduct, and striatum.^[39,40]^ PTN patients also manifest differences in diffusion tensor imaging, functional MRI, magnetic resonance spectroscopy, and other functional MRI sequences. Including the above sites and sequences in radiomics studies^[24,41,42]^ is also a meaningful attempt. Third, other potentially relevant clinical indicators were not included, such as the type of responsible vessel. Additionally, as this study used the patient’s symptomatic nerve and normal nerve for comparison, variables like the patient’s gender, age, and history of chronic disease were eliminated from consideration.

## 5. Conclusion

This study validates distinctions in radiomic textural features and clinical anatomical characteristics among PTN patients with or without vascular compression. It demonstrates the feasibility of constructing a model to aid in the diagnosis of PTN based on these indicators.

## Author contributions

**Conceptualization:** Hongjian Li.

**Data curation:** Hongjian Li, Chuan Zhang, Libing He, Ting Lei.

**Formal analysis:** Hongjian Li, Wei Yan.

**Funding acquisition:** Hanfeng Yang.

**Investigation:** Hongjian Li, Xiran Shi.

**Methodology:** Hongjian Li, Zeyong Li, Ying Liu.

**Software:** Xu Lang.

**Writing—original draft:** Hongjian Li, Qimin Yang.

**Writing—review & editing:** Hongjian Li, Baijintao Sun, Anup Bhetuwal.

## Supplementary Material


